# Annotations

**Published:** 1892-07-30

**Authors:** 


### Annotations.

**Health and Beauty.**

"Have you noticed," said a gilded youth in Hyde Park, "how much taller the girls have been these last few seasons than formerly?" The remark was just, and, though it did not seem to strike the critic, the reason for the change is obvious enough. These tall girls, with their graceful figures and free movements, have had an education in gymnastics which has developed them to a point of physical perfection of which their mothers never dreamed. A recent critic complained that little women had "gone out" in fiction, and perhaps this has happened, because to a great extent the little woman has gone out in real life. So much the better, for though in stories, and perhaps in courting days, there is something fascinating in the heroine's bird-like flutterings, in her nestling timidly in the hero's protecting arms, or standing on tip-toe to fasten a flower in his buttonhole, the charming fairy was apt, if you only knew it, to become an invalid in maturer life, and spend half her time upon the sofa. The lady who reclines on a sofa has also vanished from fiction, at least as an interesting and attractive character, and to die of consumption is no longer romantic. Health is in fashion, in the park and elsewhere. Sometimes, indeed, exercise and active amusements bulk too largely in a young girl's life, and elderly ladies complain that they have ceased to be domesticated. Certainly the average girl would resent the suggestion that the sweeping of a room is as good exercise as a game at lawn tennis, but even if she derides the notion, let her go. Domestic work may need some skill and practice, but it is not of so scientific a nature that a woman cannot learn it for herself when the need arises. That is, if she be a strong woman; your delicate fairy can only sit down and weep at her own helplessness. The eye that can see dust in its lurking-place, and the hand that can make a modest meal appetising, are doubtless desirable enough, but it is not dreaming impossible things of English women to believe that these things may be attained when the time for acquiring physical strength has passed, and the wear and tear of life has begun. Meanwhile, with women cricketers, women shooters, women bicyclists, the era of strength has come, and no one—not even the reviewer who mourns the loss of the little woman—seems to think that therewith the era of beauty has passed away.

**"Below Par."**

Curious and interesting are the weather-charts given in newspapers, the maps of the progress of epidemics in medical journals, the records of the sphygmograph, and any other attempt to render visible to the eye by diagram things which are really perceived by other senses or by the understanding only. It would be very valuable if some such chart could be given of the variation in health of different people. We should require a long table, numbered, say, from ten to a hundred, though no one person would vary much beyond ten degrees on either side of his average line. But the average varies so much with one and another. To be "well" means a very different thing for an athlete in the prime of youth from what it does for an aged man. The first considers himself "below par" if he cannot walk twenty miles without exhaustion; the second would be surprised at his own energy if he managed five. And each would be justified in his opinion and in the satisfaction or anxiety it would arouse in him. Allow that the old man is not as strong as the young, it does not follow that he is not quite as well. Judging him by his own health-line—his average condition—he may even be better. There is always a danger in getting below one's average; it implies a weakening of the system, a joint in the armour, by which the arrow of disease may enter. One who keeps pretty constantly at his "frail ordinar," as the old Scotch folk term it, is really more secure than an apparently stronger man who has fallen "below par." It is a happy phrase this, which we have borrowed from the Stock Exchange, to describe our physical condition. At "par" we are sent out into the world; not always worth as much physically as our neighbours—there are five-shilling shares as well as five-pound ones—but of a certain definite value which, by careful living, we may increase, or, by physiological extravagance, depreciate. Whether we shall rise or fall in the health market largely depends, at least after childhood, on ourselves, and we all know people who have increased their capital tenfold, and others who have wasted in thirty years a provision ample for seventy. Over-work does it now and then, but over-indulgence in what some call pleasure does it ten times oftener. And no one does it in ignorance; brain, nerves, and stomach are his bankers, warning him duly of any over-drafts he makes. We have said that it would be curious to see the health lines of different people; but, after all, it is not important. It is important only to know our own; and as the above-named monitors keep us duly informed of this, it is our own fault if we ever get "below par."

**A Fossil Institution.**

Lord Aberdeen has the unique distinction of being the President of a fossil institution, that is, of one which has no sense of its own dignity, and no touch with the best features of the day and generation in which it exists. The system of administration at the Royal Hospital for Incurables, Putney, has been condemned in plain language by the Lords' Committee. A special meeting of the governors, to which we are informed the whole of the governors were not invited by a personal notice, and the majority of whom were and are ignorant of the fact of its being called, was held on the 22nd inst. The proceedings of this meeting were extraordinary. A long statement prepared by the Committee was read, which has not been sent to the press, and which it was impossible to follow. Despite Lord Aberdeen's request for a searching, thorough, and independent inquiry, the Committee simply formulated a resolution expressing confidence in themselves. To this one of the governors, Captain Webb, moved an amendment referring the recommendation of the House of Lords' Committee to a special Committee of Governors, in conference with the Board. We have said that no adequate notice was given to the governors of the meeting; those who came were chiefly ladies, and so the amendment was lost by a large majority. This Hospital for Incurables has been reduced to its present condition owing to the fact that its funds are abundant. Captain Webb and his thirty-two supporters must promptly confer with the President, Lord Aberdeen, who has been treated with scant courtesy by the Committee, and steps must be concerted to bring the whole of the facts to the notice of every governor, to summons a second special general meeting, and to replace the present Board of Management, or at any rate to substitute some active and intelligent men for a majority of the present members. Lord Aberdeen, we believe, possesses the noble ambition to fill the position left vacant by the death of Lord Shaftesbury. Whether this be so or not, as a member of the House of Lords, and as President of the Royal Hospital for Incurables, the public, and the more intelligent of the governors look to Lord Aberdeen to secure an immediate and exhaustive inquiry into the administration of this institution. We would remind him that the Lords' Committee found the authorities of this hospital "to be incapable of effecting reforms, and extremely resentful of external observation." This was surely a prophetic utterance in view of the proceedings at the special meeting on the 22nd inst.

